# Microstructure of Whole Wheat versus White Flour and Wheat-Chickpea Flour Blends and Dough: Impact on the Glycemic Response of Pan Bread

**DOI:** 10.1155/2020/8834960

**Published:** 2020-10-05

**Authors:** Tasleem A. Zafar, Ahmed Aldughpassi, Abdulwahab Al-Mussallam, Amani Al-Othman

**Affiliations:** ^1^Department of Food Science & Nutrition, College of Life Sciences, Kuwait University, Kuwait; ^2^Department of Chemical Engineering, College of Engineering and Petroleum, Kuwait University, Kuwait; ^3^Department of Computer Science, Kuwait Institute for Scientific Research, Kuwait

## Abstract

Whole foods are generally considered healthier choices compared to processed foods. For nutritional consideration, whole wheat bread is recommended over the white bread. However, it has a similarly high effect on glycemic response (GR) as the white bread. This study is aimed at assessing the microstructure of whole wheat flour (WWF), white flour (WF), chickpea flour (BF), their blends, and dough and the GR of the bread made thereof. Scanning electron microscope analysis showed clear distinctions in the microstructure of the three flours. WWF particle size distribution had the widest spread with a polydispersity index (PDI) of 1.0 (±0.0) and wider average diameter, with *z* value of 1679.5 (±156.3) compared with the particle size of 658.9 (±160.4) and PDI of 0.740 (±0.04) for WF followed by BF with the particle size of 394.1 (±54.9) and PDI of 0.388 (±0.07) (*p* < 0.05). The falling number was significantly (*p* < 0.05) lower for WWF compared to WF or BF, indicating higher alpha-amylase activity. Thus, bread made from WWF without BF substitution exhibited a higher glycemic response similar to the bread made from WF. When partly replaced with BF, the GR of the bread made with WWF or WF reduced significantly (*p* < 0.05) in healthy individuals.

## 1. Introduction

Wheat bread is a basic staple in the diet of people around the world and a major protein source for the less affluent population. Therefore, majority of research has been dedicated to the improvement of its amino acid profile [1–4]. Relatively less attention is given to the starch, which is a major component in flour that controls the physical characteristics of dough and baked products made thereof. Furthermore, it is the starch quality that determines the effect on glycemic response (GR) of the bread.

Wheat bread is classified as a high glycemic index (GI) food. Rapid elevations in blood glucose after consuming high GI foods more frequently may cause metabolic stress, specifically associated with insulin resistance and type 2 diabetes [5]. Complex carbohydrates, including whole-grain products, because of their higher fiber and other nutrient content, are recommended for achieving better health status. Nevertheless, when it comes to wheat bread, no difference has been demonstrated in the GE of the refined white flour bread (WB) and whole wheat bread (WWB) [6–9]. The average GI of bread, based on 13 studies, made from whole wheat or white flour was equal to 71 ± 2 [8, 9].

The research to date defines GI of a carbohydrate food as a postprandial property determined by the rate of digestion and absorption. Nevertheless, the glycemic property of bread is a complex interaction of several preprandial factors, to name a few, the chemical structure of starch, the changes in the starch molecule during heating, the size of starch granules, particle size of flour, and the presence of interfering substances between starch and the hydrolytic enzymes. Hence, to develop a good quality wheat bread with low GR, it is imperative to understand the factors that influence the microstructure characteristics of wheat flour as well as the dough, which might be impacting the glycemic response of bread.

Starch in the bread is present in two chemical forms: amylopectin, an *α*, 1-4 linked glucose chain with branching as *α*, 1-6 linkages, and the amylose *α*, 1-4 glucose polymers rendering the starch structure a combination of amorphous and crystalline areas. Starch molecules go through the process of gelatinization when heated in the presence of water. Water enters the amorphous spaces, causing the starch granule to swell, disrupting its crystalline structure with amylose leaching out into the surrounding area. The process of heating gelatinizes the starch and cooling recrystallizes it, a phenomenon known as retrogradation. The higher crystallinity that occurs in a starch molecule will lead to lower digestibility and, therefore, to a lower GR [10].

The shape or size of starch granule also affects its digestibility and GR. The larger starch granule has a bigger surface area, with greater gelatinization tendency and a better probability of enzymatic hydrolysis [11]. The presence of lipid, protein, and fiber has been shown a reduction in GR, probably through lowering the degree of gelatinization, for example, by the formation of hydrophobic lipid-amylose complex, and through the protein as well as fiber competing for the available water hindering the water accessibility to starch granule, thus influencing starch gelatinization and hydrolysis by amylases [12, 13].

Legumes, a source of good quality proteins and dietary fiber, are low in total starch yet high in amylose starch, which can convert to resistant starch during baking, rendering it to produce low GR. Supplementing chickpea flour (BF) to wheat flour can help in decreasing the GR of wheat bread. BF-supplemented composite wheat bread has been evaluated for enhancement of the nutritional quality by many researchers, but only a few studies exist on the GR of the bread with inconsistent results [14, 15].

The present research is aimed at exploring the hypotheses that microstructure characteristics of whole wheat and refined wheat flour doughs differ and thus variably impact bread quality and its glycemic effect; also, wheat-chickpea composite blends will modulate dough attributes for the production of superior quality of bread exhibiting low glycemic effect. The objectives of the study were to assess the microstructure differences of the doughs made from WWF, WF, and their blends with chickpea flour (BF); test the preprandial differences of the doughs (if any) affecting the bread on the postprandial glycemic responses; and determine the best ratio of wheat-chickpea blend for producing a low-glycemic bread. To achieve these objectives, three experiments were conducted to measure (1) the microstructure properties of dough made from WWF and WF and (2) of the wheat-BF blends at various ratios and (3) the GR of WWB and WB alone or as composite blends with BF.

## 2. Materials and Methods

### 2.1. Experiment 1: Microstructure Characteristics of WWF and WF Doughs

#### 2.1.1. Particle Size Analysis

Both WWF and WF were procured from Kuwait Flour Mills & Bakeries Company, Shuwaikh. Commercially available BF was obtained from the local market.

Particle size analysis was performed by the dynamic light scattering method [16] using dynamic light scattering instrument (Model: ZEN3500, Malvern Instruments Ltd., UK). Each sample was analyzed in triplicate, and average values are reported as mean ± SD. The light source used was a 532 nm, 50 mW green laser. Measurements were taken at 25°, and the intensity data were processed by using the appropriate software (DTS5/nanoapplications). Essentially, the analysis model is general purpose; the instrument measures the diffusion coefficient (*D*) of the dispersed particles and evaluates the hydrodynamic radius (rh) in terms of the Stokes-Einstein equation: rh = *kT*/6*πη* *D*, where *η*, *k*, and *T* are the viscosity of the medium, the Boltzman constant, and the absolute temperature, respectively. The observed *z* value (intensity-weighted average dia (in nm), polydispersity index (PDI)), i.e., the width of particle size distribution peak, and areas under the particle size distribution peaks have been reported.

#### 2.1.2. Scanning Electron Microscope (SEM)

The microstructure of WWF and WF dough samples was evaluated using a SEM (JEOL, JSM-5410LV, Tokyo, Japan) at an accelerating voltage of 20 kV. Dough was prepared by using Farinograph Water Absorption (FWA) [17]. The flour samples were measured directly in the powder form whereas the WWF and WF doughs were first freeze dried (Virtis, model: Unitop 800L, USA), then coarsely crushed, added to cello tape for taking SEM measurements on these chunks of dried dough pieces. Each sample was coated with gold in a sputter coater (Structure Probe, West Chester, PA) before being scanned and photographed at different magnifications ranging from 250 to 5000.

#### 2.1.3. Falling Number Measurements

Falling number values of these blends were determined as per the standard AACC method 56-81B [17] with a Falling Number Apparatus (Perten Instruments, Sweden). The falling number (FN) method is a reliable fast method to determine the *α*-amylase activity of wheat flour. The higher the FN value, the lower the *α*-amylase activity and vice versa.

### 2.2. Experiment 2: Microstructure Properties of WWF : BF and WF : BF Blends and Dough

BF substituted WWF and WF in different percentages (10%, 20%, 30%, and 40%); blends were prepared by mixing them thoroughly in dry condition. Moisture content in these flours, blends, and dough was determined by the standard AACC method 44-15A [17].

Particle size analysis, SEM images, and FN measurements of the chickpea flour, flour blends prepared by BF substitution of WWF and WF, and their dough were determined by the same procedure as explained in the earlier section.

### 2.3. Experiment 3: Bread Testing

#### 2.3.1. Bread Making

Composite bread from both WWF and WF substituting BF at 0% (control), 20, 30, and 40% level was prepared using the formula given earlier [15, 18] under standard conditions of the straight dough-optimized procedure for bread making, AACC method 10-10B [17].

#### 2.3.2. Glycemic Response Test

Young healthy males and females, age 18–28 years (mean ± SD of 22.4 ± 2.4) with normal range of body mass index (kg/m^2^) of 22.8 ± 2.2, participated in the study. Subjects were recruited at the College of Life Sciences, Kuwait University, through flyers. A sample size (*n* = 15) was calculated based on power analyses from a similar study to detect significant differences among the breads with a level of *α* 0.05 and *β* 0.08 [15]. Exclusion criteria included subjects with fasting glucose > 5.6 mmol, those with diabetes or on medication affecting blood glucose concentration, breakfast skippers, and restrained eaters. Female subjects were not scheduled for a test session during their menstrual cycle to avoid any hormonal effect on blood glucose. The research protocol for the study was approved by the Health Sciences Humans' Ethics Committee of Kuwait University. All study subjects signed the informed consent form.

A randomized, single-blinded within-subject, repeated measures study design was followed. Subjects came at the same time for all test sessions between 8:30 and 10:30 am after an overnight fast. The subjects were asked to consume the preweighed bread in a randomized order on nine different visits, roughly 4-5 days apart. Subjects were advised to consume the bread and a bottle of mineral water within 10 min, while seated in individual booths. The eight experimental breads tested were prepared in the Department's Baking Laboratory, and one test bread (plain, white bread, commonly consumed by local people) was purchased for a reference from the local market (Kuwait Flour Mills and Bakeries Co.). The commercial bread was similar in nutritional composition to the experimental control bread. All the test breads were provided and consumed in an amount to supply 50 g of available carbohydrates. Blood glucose concentration was measured at baseline and then at 15, 30, 45, 60, and 90 min by a finger-prick method using a Monojector Lancet Device and a portable blood glucose monitoring system (One Touch Ultra, LifeScan Inc. and Johnson & Johnson Company, USA).

### 2.4. Statistical Analysis

All the results for the microstructure of flour and dough are expressed on a 14% moisture level. Research data were analyzed for analysis of variance using SPSS version 17 for Windows. Wherever appropriate, the mean values ± standard deviations of these samples using a significance level of *p* < 0.05 are presented.

Repeated measures analysis of variance was conducted on blood glucose concentration as a change from the baseline at each time point to assess treatment, time, and any time by treatment interaction over 90 min using two-way ANOVA, followed by Tukey's post hoc tests to identify significant mean differences among treatments at each time point of measurements. Area under the blood glucose concentration curves was analyzed using one-way ANOVA and Tukey's post hoc assessment. Data are presented as mean ± SD, and the significance level is set at *p* < 0.05.

## 3. Results and Discussion

The WWF, WF, and BF used in the study had moisture contents of 10.58, 10.54, and 7.61%, respectively.

### 3.1. Particle Size of Flour Samples

**Figure 1:**
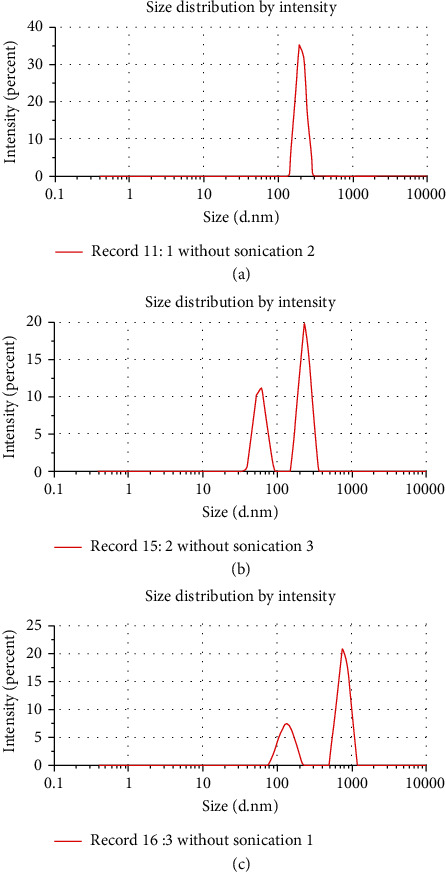
(a–c) Particle size distribution of chickpea flour (BF), wheat flour (WF), and whole wheat flour (WWF) measured by dynamic light scattering, respectively.

The distribution of the particle size of WF, WWF, and BF samples is presented in Table 1 and Figure 1. From the data in Table 1, the *z* value of WWF differed significantly (*p* < 0.05) from that of WF and BF. The higher intensity-weighted average diameter (in nm) for WWF may be due to the large particles of wheat bran [13]. Figure 1 shows that the BF sample had a very uniform particle size (single peak area) compared with the WF and WWF having two peak areas. The lowest polydispersity index value of 0.388 (±0.07) also indicates that the particle size distribution in the BF sample is much more uniform, as well as smaller, than both the WF and WWF samples. The WWF sample had the highest PDI value (1.0 ± 0.0) that indicated a wider distribution in its particle size.

### 3.2. Scanning Electron Microscope

**Figure 2:**
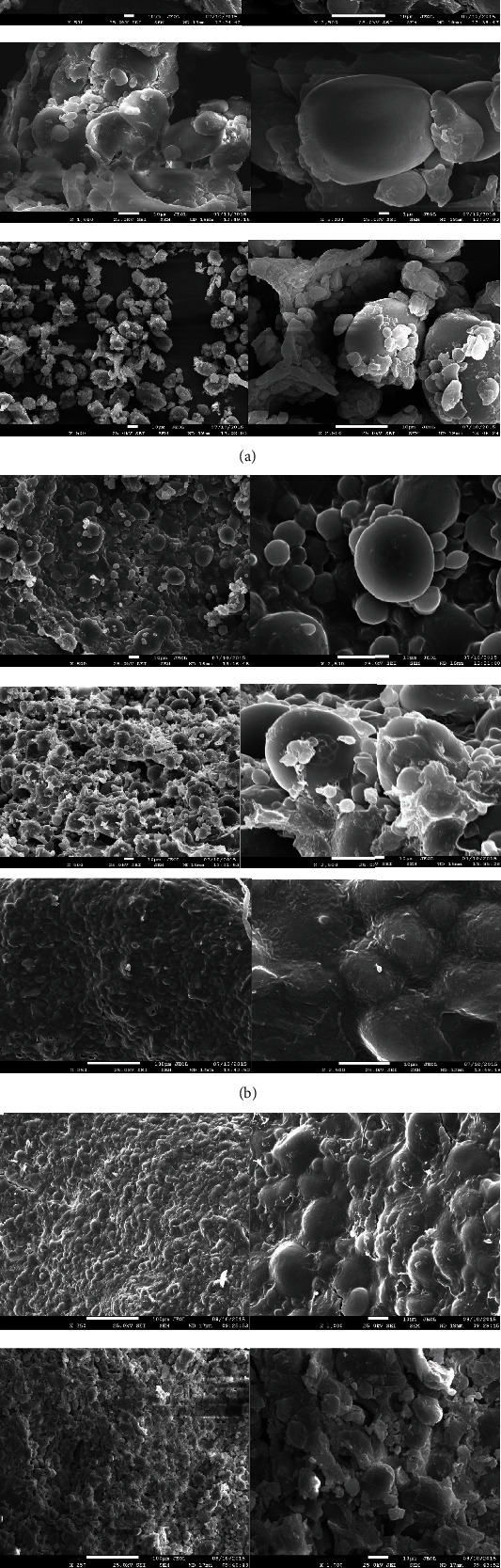
Scanning electron microscope (SEM) images of white wheat, whole wheat, and chickpea flours, doughs, and their blends. (a) SEM of flours: top, white flour; middle, whole wheat flour; bottom, chickpea flour. (b) SEM of doughs: top, white flour dough; middle, whole wheat flour dough; bottom, chickpea flour dough. (c) SEM of doughs made with wheat flour and chickpea flour blends: top, WF : BF with 60 : 40 blends; bottom, WWF : BF with 60 : 40 blends.

The scanning electron microscope images of WF, WWF, BF raw flours, their doughs, and 60 : 40 ratio flour blend doughs are presented in Figures 2(a)–2(c). These micrographs show the morphological characteristics of starch granules of wheat flour and BF as well as the development of continuous sheet-like structures covering these starch granules after dough making. The WF and WWF show two types of starch granule distributions, large granules measuring about 35 *μ*m and small granules about 2-10 *μ*m, whereas the BF shows small granules of about 10 *μ*m, but a lot of protein bodies and protein wedge-like structures (Figure 2(a): top, middle, and bottom rows). The reason for the higher number of protein bodies in BF is its higher protein content of about 27% compared to about 13-15% in wheat flour samples [19].

When the wheat flour and BF were made into their respective doughs using the optimal amount of plain distilled water, a protein sheet-like structure was developed in wheat flour doughs (Figure 2(b): top and middle rows), but the starch granules maintained their morphological identities as no heating was involved to disrupt their granular structures. Heating of dough during baking is known to gelatinize these starch granules leading to the development of a continuous matrix of gelatinized starch [12, 20]. Because of a much higher amount of protein present in the BF, a continuous sheet-like structure was developed during dough making that had enveloped almost all the starch granules of chickpea flour (Figure 2(b): bottom row). Particularly, it is apparent that at the higher ratio of 60 : 40 blend of wheat flour and BF, when the higher protein content is coming from BF, all the starch granules of wheat and chickpea were covered with a continuous sheet-like structure of proteins (Figure 2(c): top and bottom rows, respectively).

### 3.3. Falling Number

The falling number values obtained for the WF, WWF, and their blends with BF are presented in Table 2. The replacement of WF with BF significantly increased the FN value at all levels. A similar trend was observed when WWF was replaced with varying levels of BF. FN values were found to be significantly lower (*p* < 0.05) for WWF than both the WF and BF, and no significant difference was observed between WF and BF. The FN values are affected by the flour granulation, type of sprout damage (sprouting increases *α*-amylase content 1000-fold), and extent of damaged starch content of the flour samples, and this ultimately affects the quality of finished bakery products [21]. Although the BF had a finer granulation, it is known to be very low in the *α*-amylase enzyme, thus giving similar FN values to those of WF. Also, because finer particles of BF gelatinized much faster, giving higher viscosity in the FN apparatus, this leads to higher FN value (Table 2).

The FN value for obtaining desirable loaf volume in pan bread must be in the range of 250 to 300, which is usually obtained by adding *α*-amylase sources, like cereal malt and bacterial or fungal *α*-amylases. Our major objective here in this study was not about the loaf volume of pan bread but to examine the effect of chickpea flour on the glycemic properties of breads prepared from these blends. The use of a faster FN method is aimed at not only finding the *α*-amylase activity status of WF : BF and WWF : BF blends but also examining if the BF addition inhibits the *α*-amylase activity in these blends by examining the FN values. Higher FN values obtained indicated the inhibition of *α*-amylase activity when the level of BF was increased in these blends. With this lower *α*-amylase activity in chickpea : wheat flour blends, only the pan bread specific loaf volume was lowered as compared with the control WF or WWF samples, but sensory quality was not adversely affected. A number of studies have shown that chickpea flour has proteinaceous fractions which inhibit the *α*-amylase activity. Even the phenolic compounds present in bean flours, including chickpea flour, have been shown to exhibit enzyme inhibitory activities providing benefits to the individuals suffering from hyperglycemia and hypertension [22]. Ercan and El-Nehir [23] have shown for the first time the presence of *α*-amylase and lipase inhibitors present in chickpeas as well as in flour beetles. Wang et al. [24] have shown that the proteins with a cupin domain are the new *α*-amylase inhibitors present in chickpeas. Akhtar et al. [25] have characterized the role of certain polysaccharides present in chickpeas for inhibiting the *α*-amylase activity and suggested their use as a hypoglycemic agent for managing type 2 diabetes. In another recent study, Zou et al. [26] have shown the presence of proteinaceous *α*-amylase inhibitors which slow down the starch digestion in pasta products. Thus, the use of FN value measurements was aimed at corroborating our viewpoint of slowing down starch digestion through the inhibition of *α*-amylase activity for producing lower glycemic effects of feeding wheat flour : chickpea flour breads.

### 3.4. Glycemic Response Test

**Figure 3:**
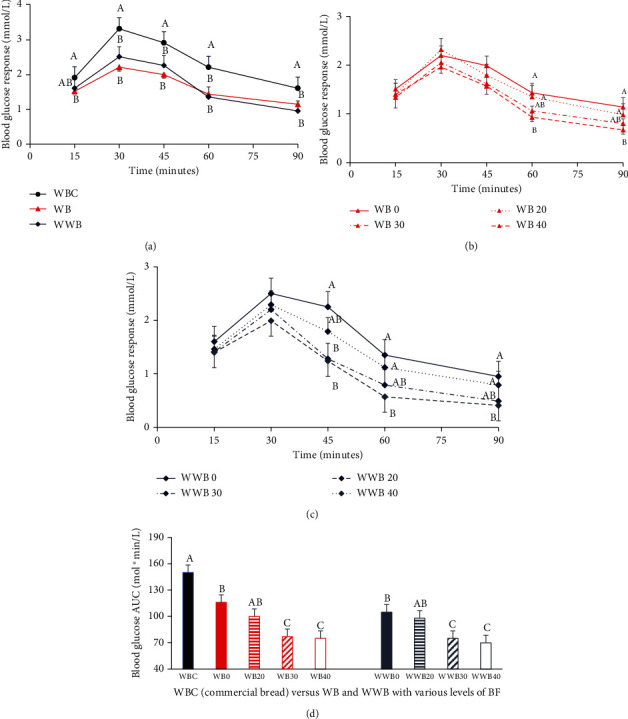
Effect of bread consumption on blood glucose concentration over time for 90 minutes in healthy volunteers. (a) Blood glucose concentration response to white bread, whole wheat bread, and wheat bread purchased from the market. (b) Blood glucose concentration in response to chickpea flour substituted at various ratios to white bread. (c) Blood glucose concentration in response to chickpea flour substituted at various ratios to whole wheat bread. (d) Blood glucose concentration under the area under the curve (AUC) in response to chickpea flour substituted at various ratios to white flour bread and whole wheat bread compared to commercial wheat bread. Data is presented as mean ± SEM (*n* = 15). (a–c) Values within the same time points are significant at *p* < 0.05. (d) Different superscript letters on the bars represent differences among the breads at *p* < 0.05. Legends: WBC = wheat bread commercial (from the supermarket); WB = white bread; WWB = whole wheat bread; WB/WWB 0, 20, 30, and 40 refer to chickpea flour substitution at 0 (control), 20%, 30%, and 40% level to white flour or whole wheat flour.

Blood glucose changes measured over 90 min and the area under the curves calculated are presented in Figure 3. The glycemic response followed a similar pattern by both series of bread from WWF and WF controls, their composite blends with BF, and the commercial bread, which was used for comparison. The maximum change in blood glucose level observed in all breads was at about 30 min, which steadily declined after that till the end of the experiment. It is not surprising to note that no differences were recorded in the blood glucose concentrations at any time point between the two control breads made from WWF and WF, as other workers also declared no difference in the GR of bread made with whole wheat or refined wheat flour [6, 8, 9]. However, both the lab-prepared breads were significantly lower than the commercial bread at all time points (Figures 3(a) and 3(d)).

The lack of difference between the control breads on the GR can be explained by the physical differences in the microstructure characteristics of WWF and WF, which eliminated the postprandial differences in the GR. For instance, the larger particle size of WWF as evidenced by significantly higher *z* value of 1679.5 (±156.3) versus 658.9 (±160.4) for WF (Table 1) has rendered WWF highly susceptible to enzymatic hydrolysis during dough development and bread-making stages [27]. The evidence of the impact of the microstructural differences of both flours further strengthened by the lower FN of 517 (±26) for WWF versus 691 (±2.8) for WF (Table 2) suggests increased hydrolysis of the WWF starch due to the higher amylase accessibility of WWF starch particles compared to the WF particles. The alpha-amylase, found in the wheat grain's aleurone layer, is retained in WWF, but not in WF, refined wheat flour [28]. The effect of amylases on starch hydrolysis has been well recognized in the food industry, including the bread industry [29].

Supplementation with *α*-amylases is a common practice for the standardization of flour for their antistaling and loaf volume enhancement effects in commercial bread making [25]. Beta-amylase abundantly occurs naturally in wheat flour, but is quickly inactivated during baking before the starch is fully gelatinized, whereas the desirable hydrolytic products, such as limit dextrin, maltose molecules, and monosaccharides, are continuously released by alpha-amylases during the first stage of baking [30, 31]. A mixture of these hydrolytic products [31, 32] is beneficial for enhancing the physical qualities such as softness and volume of the bread, yet they convert the whole-wheat complex carbohydrates into quickly absorbable simple carbohydrates, which raise the glycemic effect of the bread. The similar outcome of WWF and WF on GR in the present study (Figures 3(a) and 3(d)) or reported elsewhere [6, 8, 9] might be because of the amylases that are present naturally in the whole wheat flour or are added from an exogenous source, as is evidenced by the significantly higher GR produced by the commercial bread used for comparison in the present study (Figures 3(a) and 3(d)).

Chickpea flour substitution became effective but only at the higher levels of 30-40% in both series of breads, similarly, compared to the control breads (without BF) after the 45- to 60-minute time period (Figures 3(b) and 3(c)). The microstructural alterations of the WWF, WF, their respective doughs, and the composite dough at the BF ratio of 60 : 40 demonstrated the morphological changes in Figures 2(a)–2(c). The covering of all starch granules by the presence of higher protein content in BF, which masked the enzymatic accessibility of starch molecules and thus limited the preprandial starch hydrolysis, is demonstrated physiologically by the postprandial decrease in the GR of the wheat flour.

The ample body of literature available on the postprandial glycemic responses of carbohydrate foods, affected by factors that modify the starch structure and its accessibility to digestive enzymes, is at the gut level. For example, the development of resistant starch through recrystallization of the starch molecule via the process of retrogradation, formation of amylose-lipid complex, presence of protein or fiber, etc., all limit *in vivo* starch hydrolysis and the release of glucose into the blood circulation [10, 13, 21]. The current study has, however, highlighted that the microstructure properties of the whole wheat flour because of the large surface area of the starch granules available for enzymatic action have an impact on the glycemic response of the bread, presumably through the preprandial amylolytic products, such as simple sugars from carbohydrate hydrolysis. Furthermore, chickpea flour could modulate the high GR of the wheat bread (both WB and WWB) by masking starch molecules with protein layers causing interference in the starch hydrolysis by alpha-amylases, present naturally in the wheat flour.

## 4. Conclusions

This study is the first to our knowledge that has compared the microstructure properties of whole wheat flour versus refined wheat flour doughs and their breads on the *in vivo* glycemic responses. The average larger particle size and the widest spread of WWF reflected in significantly lower falling number values, indicating high starch accessibility for its excessive *α*-amylase activity than either of the WF and BF. Thus, the higher hydrolytic products of starch from whole wheat, supposedly complex carbohydrate with higher proportion of fiber, converted the WWB into high-glycemic bread similar to the bread from white flour, a refined carbohydrate. The lower glycemic response of the higher BF : wheat flour composite breads (40 : 60 levels) may be attributable to the coating of starch granules by the presence of a high amount of protein and fiber in BF, as evidenced by the scanning electron microscope results.

Health professionals recommending whole wheat bread for its high fiber and other nutrient content need caution in suggesting it to the people with diabetes, because patients with diabetes, considering WWB as a better option over the white bread, may consume it excessively and bring their blood glucose regulations at a halt. From these data, wheat-chickpea composite bread would be a better choice than plain whole wheat bread for controlling blood glucose levels within the normal range. Future research focused on the microstructural characteristic modulated by hydrolytic enzymes, natural or supplemented, and other additives to the wheat flour on the glycemic response of bread in people with or without diabetes is warranted, in order to succeed in developing high-quality staple pan bread with the low glycemic index.

## Figures and Tables

**Table 1:** Particle size of wheat and chickpea flour samples measured by dynamic light scattering.

Sample measured	*z* value, intensity-weighted average diameter (nm)	Polydispersity index (PDI), width of distribution	Peak 1 area intensity (%)	Peak 2 area intensity (%)
BF	394.1 ± 54.9^a^	0.388 ± 0.07^a^	100.0 ± 0.0^a^	0.0 ± 0.0^a^
WF	658.9 ± 160.4^a^	0.740 ± 0.04^b^	59.7 ± 3.9^b^	40.3 ± 3.9^b^
WWF	1679.5 ± 156.3^b^	1.00 ± 0.0^c^	66.3 ± 3.7^b^	33.7 ± 3.7^b^

The data is presented as mean ± SD. The values with different superscripts in a column differ significantly at *p* ≤ 0.05. Legends: BF: chickpea flour; WF: white flour; WWF: whole wheat flour.

**Table 2:** Falling number values of the white flour, whole wheat flour, chickpea flour, and their blends.

	Falling number (seconds)	
WF/WWF : BF	WF	WWF
100 : 0	691 ± 2.8^a^	517 ± 26.2^a^
90 : 10	826 ± 4.9^b^	596 ± 28.3^b^
80 : 20	811 ± 18.4^b^	589 ± 4.9^b^
70 : 30	753 ± 0.7^c^	563 ± 7.8^ab^
60 : 40	756 ± 24.7^c^	591 ± 31.1^b^
0 : 100	679 ± 0.7^a^	679 ± 0.7^c^

The data is presented as mean ± SD. The values for WF/WWF and BF blends with different superscript letters within a column differ significantly at *p* ≤ 0.05. Legends: WF: white flour; BF: chickpea flour; WWF: whole wheat flour.

## Data Availability

The data obtained from the research is reported in Results and Discussion in the form of “tables and figures.” If needed, the processed data could be made available upon request.
